# Spectrocolorimetric assessment of cartilage plugs after autologous osteochondral grafting: correlations between color indices and histological findings in a rabbit model

**DOI:** 10.1186/ar2287

**Published:** 2007-09-10

**Authors:** Koji Hattori, Kota Uematsu, Yohei Tanikake, Takashi Habata, Yasuhito Tanaka, Hiroshi Yajima, Yoshinori Takakura

**Affiliations:** 1 Department of DAIWA HOUSE Indoor Environmental Medicine, Nara Medical University, Kashihara, Nara, Japan; 2 Department of Orthopaedic Surgery, Nara Medical University, Kashihara, Nara, Japan

## Abstract

We investigated the use of a commercial spectrocolorimeter and the application of two color models (L* a* b* colorimetric system and spectral reflectance distribution) to describe and quantify cartilage plugs in a rabbit model of osteochondral autografting. Osteochondral plugs were removed and then replaced in their original positions in Japanese white rabbits. The rabbits were sacrificed at 4 or 12 weeks after the operation and cartilage samples were assessed using a spectrocolorimeter. The samples were retrospectively divided into two groups on the basis of the histological findings (group H: hyaline cartilage, successful; group F: fibrous tissue or fibrocartilage, failure) and investigated for possible significant differences in the spectrocolorimetric analyses between the two groups. Moreover, the relationships between the spectrocolorimetric indices and the Mankin histological score were examined. In the L* a* b* colorimetric system, the L* values were significantly lower in group H than in group F (*P *= 0.02), whereas the a* values were significantly higher in group H than in group F (*P *= 0.006). Regarding the spectral reflectance distribution, the spectral reflectance percentage 470 (SRP_470_) values, as a coincidence index for the spectral reflectance distribution (400 to 470 nm in wavelength) of the cartilage plugs with respect to intact cartilage, were 99.8 ± 6.7% in group H and 119.8 ± 10.6% in group F, and the difference between these values was significant (*P *= 0.005). Furthermore, the a* values were significantly correlated with the histological score (*P *= 0.004, r = -0.76). The SRP_470 _values were also significantly correlated with the histological score (*P *= 0.01, r = 0.67). Our findings demonstrate the ability of spectrocolorimetric measurements to predict the histological findings of cartilage plugs after autologous osteochondral grafting. In particular, the a* values and SRP_470 _values can be used to judge the surface condition of an osteochondral plug on the basis of objective data. Therefore, spectrocolorimetry may contribute to orthopedics, rheumatology and related research in arthritis, and arthroscopic use of this method may potentially be preferable for *in vivo *assessment.

## Introduction

Although articular cartilage shows durability and the ability to maintain itself, it has limited capacity for repair [1,2]. The repair cartilage that forms as a result of articular injury has a different structure from hyaline cartilage and exhibits inferior mechanical properties and wear characteristics. Thus, once damage has occurred, it continues to accumulate, eventually leading to complete loss of the articular surface and exposure of the underlying bone. These changes are almost always associated with severely impaired joint function and clinical symptoms of redness, swelling and pain [1,3,4]. Therefore, the poor quality of cartilage repair tissue has led surgeons to develop procedures intended to improve articular cartilage repair, thereby improving joint function and decreasing joint pain. Several surgical techniques are currently used in clinical practice, namely debridement, microfracture, drilling, abrasion arthroplasty, autologous osteochondral grafting (OCG) and cultured autologous chondrocyte transplantation [3-7].

OCG has become popular as a means for treating articular cartilage defects [5,8]. This technique involves transplantation of osteochondral plugs from a non-weightbearing region to the defect lesion. OCG has several advantages over other surgical treatments for articular cartilage defects. Specifically, OCG is currently the only technique able to fill a joint surface defect with hyaline cartilage, is a relatively simply method compared with autologous chondrocyte transplantation, shows little immunological rejection and is disease-free. However, 5% to 20% of the procedures fail overtly, and several authors have noted the presence of fibrillation or fibrocartilage formation in patients on later histological examination [8-11]. Fibrillation or conversion to fibrocartilage is considered undesirable because these tissues are less smooth and less stiff than normal cartilage and may tend to slough over time. However, the fibrous overgrowth is grossly similar in appearance to normal articular cartilage, and histological differences may not be obvious in subsequent arthroscopic evaluation [11].

We have investigated the use of a commercial spectrocolorimeter and the application of two color models (L* a* b* colorimetric system and spectral reflectance distribution) to describe and quantify articular cartilage. Previously, we measured the colors of rabbit knee cartilage using a spectrocolorimeter. However, no studies have yet focused on spectrocolorimetric and histological assessment of cartilage plugs after OCG. The purpose of the present study was to determine the efficacy of a spectrocolorimeter for evaluating OCG. To this end, we quantitatively evaluated osteochondral cartilage plugs using an experimental rabbit model.

## Materials and methods

### Experimental model: rabbit OCG model

The Animal Research Committee of Nara Medical University approved this investigation. Fifteen adult Japanese white rabbits underwent the following surgical procedure under anesthesia with ketamine (35 mg/kg intramuscularly) and xylazine (7 mg/kg intramuscularly). The rabbits were placed in the supine position and surgery was performed on the left knee. After shaving and sterile prepping of the lower limb, an anteromedial arthrotomy was performed in the left knee. The patella was dislocated laterally and the patellar groove was exposed. OCG was performed in the patellar groove. A full-thickness cylindrical osteochondral plug (5 mm in diameter, 5 mm in depth) was harvested using an Osteochondral Autograft Transfer System (OATS; Arthrex, Naples, FL, USA), and subsequently returned to its original position, such that the articular surface of the plug was flush with the surrounding native articular cartilage. The knee wound was irrigated with saline solution and closed in layers with 2-0 vicryl sutures. No cast was applied to the lower leg. The right knee was left without treatment as a control. The rabbits were sacrificed with an overdose of phenobarbital sodium salt at 4 or 12 weeks after the operation.

### Macroscopic evaluation and scoring

After sacrifice, each knee joint was opened and dissected free from all the soft tissues before the tibia was removed. The cartilage surfaces were observed with the naked eye and photographed. Each cartilage plug was graded for its gross appearance according to Moran and colleagues [12] (Table 1) by one blinded observer (TH).

**Table 1:** Modified Moran's scoring system

Category	Grade
I. Intra-articular adhesion	
None	2
Minimal (fine, loose fibrous tissue)	1
Major (thick, dense fibrous tissue)	0
	
II. Restoration of articular surface contour	
Complete	2
Partial	1
None	0
	
III. Erosion of cartilage	
None	2
Graft only	1
Graft and adjacent normal cartilage	0
	
IV. Appearance of cartilage	
Translucent	2
Opaque	1
Discolored or irregular	0

### Spectrocolorimetric measurements

Articular cartilage evaluation was performed using a commercial spectrocolorimeter (X-Rite SP64; X-Rite KK, Tokyo, Japan) driven by a software program (Color/Reader I; Color Techno System Corp., Tokyo, Japan). The reference illumination was D 65 (standard daylight), the geometry was d/8, the incident light was diffuse and the observation angle was 10°, according to the Commission Internationale d'Eclairage (CIE) 15.2 publication and International Organization for Standardization (ISO) 7724/1 recommendations. The X-Rite SP64 was positioned with minimal pressure perpendicular to the object and the data were reported in the L* a* b* colorimetric system and the spectral reflectance distribution of the object's color. The L* a* b* colorimetric system is currently the most widely used system for color analysis and is composed of three coordinates, namely color lightness and two coordinates related to chromatic components. The L* (luminance) value measures brightness ranging from black (0) to white (100), while the a* value expresses the color spectrum from green (-) to red (+) and the b* value expresses the color spectrum from blue (-) to yellow (+). Regarding the other index of cartilage color evaluation, the spectral reflectance distribution was automatically calculated at 10 nm wavelength intervals from 400 to 700 nm.

**Figure 1:**
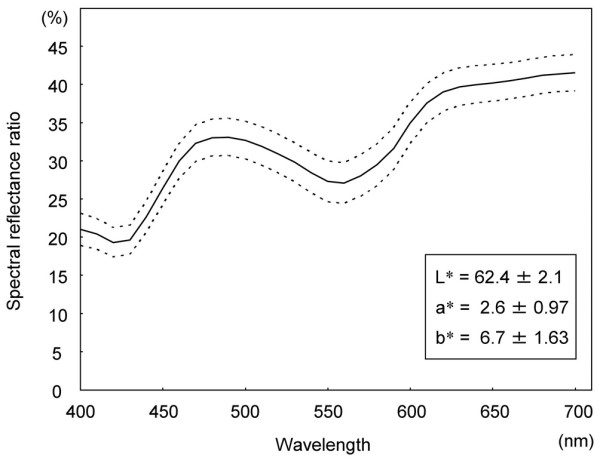
Standard L*, a* and b* values (mean ± standard deviation) and standard spectral reflectance curve (thick line, mean; broken lines, ± standard deviation) of Japanese white rabbit cartilage (*n *= 19).

The X-Rite SP64 had a measuring area of 4 mm in diameter. To calibrate the instrument, a standard white plate and black trap were used. The X-Rite SP64 was positioned with minimal pressure perpendicular to the cartilage plug or the intact cartilage area as a control. Three consecutive measurements of the L*, a* and b* values and the spectral reflectance ratio per site were averaged for each cartilage measurement. Previously, we determined the standard L*, a* and b* values and the standard spectral reflectance ratio of intact articular cartilage in Japanese white rabbits (Figure 1). As a coincidence index for the spectral reflectance distribution of the cartilage plug after OCG with respect to intact cartilage, the spectral reflectance percentage (SRP) was determined. The SRP_700 _values between 400 and 700 nm in wavelength and the SRP_470 _values between 400 and 470 nm in wavelength were expressed using the following equations:

SRP700=∫400700f(x)dx/∫400700g(x)dx×100(%)
 MathType@MTEF@5@5@+=feaafiart1ev1aaatCvAUfKttLearuWrP9MDH5MBPbIqV92AaeXatLxBI9gBaebbnrfifHhDYfgasaacH8akY=wiFfYdH8Gipec8Eeeu0xXdbba9frFj0=OqFfea0dXdd9vqai=hGuQ8kuc9pgc9s8qqaq=dirpe0xb9q8qiLsFr0=vr0=vr0dc8meaabaqaciaacaGaaeqabaqabeGadaaakeaaieaacqWFtbWucqWFsbGucqWFqbaudaWgaaWcbaGaeG4naCJaeGimaaJaeGimaadabeaakiabg2da9maapedabaGaemOzayMaeiikaGIaemiEaGNaeiykaKcaleaacqaI0aancqaIWaamcqaIWaamaeaacqaI3aWncqaIWaamcqaIWaama0Gaey4kIipakiabdsgaKjabdIha4jabc+caVmaapedabaGaem4zaCMaeiikaGIaemiEaGNaeiykaKcaleaacqaI0aancqaIWaamcqaIWaamaeaacqaI3aWncqaIWaamcqaIWaama0Gaey4kIipakiabdsgaKjabdIha4jabgEna0kabigdaXiabicdaWiabicdaWiabcIcaOiabcwcaLiabcMcaPaaa@5ADF@

SRP470=∫400470f(x)dx/∫400470g(x)dx×100(%)
 MathType@MTEF@5@5@+=feaafiart1ev1aaatCvAUfKttLearuWrP9MDH5MBPbIqV92AaeXatLxBI9gBaebbnrfifHhDYfgasaacH8akY=wiFfYdH8Gipec8Eeeu0xXdbba9frFj0=OqFfea0dXdd9vqai=hGuQ8kuc9pgc9s8qqaq=dirpe0xb9q8qiLsFr0=vr0=vr0dc8meaabaqaciaacaGaaeqabaqabeGadaaakeaaieaacqWFtbWucqWFsbGucqWFqbaudaWgaaWcbaGaeGinaqJaeG4naCJaeGimaadabeaakiabg2da9maapedabaGaemOzayMaeiikaGIaemiEaGNaeiykaKcaleaacqaI0aancqaIWaamcqaIWaamaeaacqaI0aancqaI3aWncqaIWaama0Gaey4kIipakiabdsgaKjabdIha4jabc+caVmaapedabaGaem4zaCMaeiikaGIaemiEaGNaeiykaKcaleaacqaI0aancqaIWaamcqaIWaamaeaacqaI0aancqaI3aWncqaIWaama0Gaey4kIipakiabdsgaKjabdIha4jabgEna0kabigdaXiabicdaWiabicdaWiabcIcaOiabcwcaLiabcMcaPaaa@5AF7@

y = f(x), y = g(x), x: wavelength, y: reflectance ratio

where f(x) is the numerical formula of the cartilage plug sample in the spectral reflectance graph and g(x) is the numerical formula of the intact cartilage sample (standard) in the spectral reflectance graph (Figure 1).

### Histological evaluation and scoring

After the spectrocolorimetric evaluation, the specimens were fixed in 10% neutral-buffered formalin, decalcified in 0.25 mol/l EDTA in phosphate-buffered saline, dehydrated through a graded ethanol series and embedded in paraffin wax. Sagittal sections (5 μm thick) were cut and then stained with hematoxylin and eosin, toluidine blue and Safranin-O fast green. Each section was graded using the histological scale described by Mankin and colleagues [13] (Table 2). Histological assessments were performed by one blinded observer (YT). Cartilage samples were divided into two groups on the basis of the histological findings of the plug surface by another blinded observer (TH) as follows: group H, the cartilage plug retained the features of hyaline cartilage; group F, the cartilage plug consisted of fibrous tissue and/or fibrocartilage. The samples were retrospectively divided into the two groups and investigated for possible significant differences in the spectrocolorimetric analyses between the two groups.

**Table 2:** Mankin's histological histochemical grading

Category	Grade
I. Structure	
Normal	0
Surface irregularities	1
Pannus and surface irregularities	2
Clefts to transitional zone	3
Clefts to radial zone	4
Clefts to calcified zone	5
Complete disorganization	6
	
II. Cells	
Normal	0
Diffuse hypercellularity	1
Cloning	2
Hypocellularity	3
	
III. Safranin-O staining	
Normal	0
Slightly reduced	1
Moderate reduced	2
Severe reduced	3
No dye noted	4
	
IV. Tidemark integrity	
Intact	0
Crossed by blood vessels	1

### Statistical analysis

Differences among spectrocolorimetric data were analyzed using the non-parametric Mann-Whitney U-test. The relationships between spectrocolorimetric data and the histological score were analyzed using the non-parametric Spearman's rank-order correlation method. The significance level was set at *P *< 0.05.

## Results

### Macroscopic findings

Gross inspections were performed at 4 and 12 weeks after the operation. OCG displayed similar cartilage to the surrounding cartilage at both intervals. The margin around each osteochondral plug was a little faint but still detectable. The surface of the cartilage plugs appeared to be almost smooth. All of the surfaces were glistening and white in appearance. From the macroscopic findings, no histological differences could be detected among the cartilage plugs. The mean macroscopic scores were 5.8 points at 4 weeks and 5.9 points at 12 weeks. There was no significant difference between the macroscopic scores at 4 and 12 weeks (*P *= 0.54).

### Spectrocolorimetric findings

Color measurements were carried out on the cartilage plugs and the control cartilage (patella groove of the right knee). The differences in the L*, a* and b* values of the cartilage plugs at 4 and 12 weeks after the operation and the control cartilage are shown in Table 3. There were no significant differences in the L*, a* and b* values at 4 and 12 weeks after the operation. Compared to the control cartilage, the cartilage plugs at 4 weeks had significantly lower b* values (*P *= 0.004) and the cartilage plugs at 12 weeks had significantly lower L* and b* values (*P *= 0.02 and *P *= 0.003, respectively).

**Table 3:** L* a* b* color change of the plug cartilage at 4 and 12 weeks after osteochondral autograft

Parameter	4 weeks	12 weeks	*P *value^a^
L* value	58.3 ± 3.9	59.5 ± 2.2^b^	NS
L* value/control	62.7 ± 2.8	62.4 ± 1.6	
			
a* value	3.0 ± 1.3	3.3 ± 0.9	NS
a* value/control	2.2 ± 1.0	2.4 ± 0.9	
			
b* value	-0.8 ± 1.6^b^	0.1 ± 2.0^b^	NS
b* value/control	6.1 ± 1.1	6.6 ± 1.6	

Data are mean ± standard deviation. ^a^*P *values based on Mann-Whitney U-test, 4 weeks versus 12 weeks. ^b^*P *< 0.05 versus control. NS, not significant.

**Figure 2:**
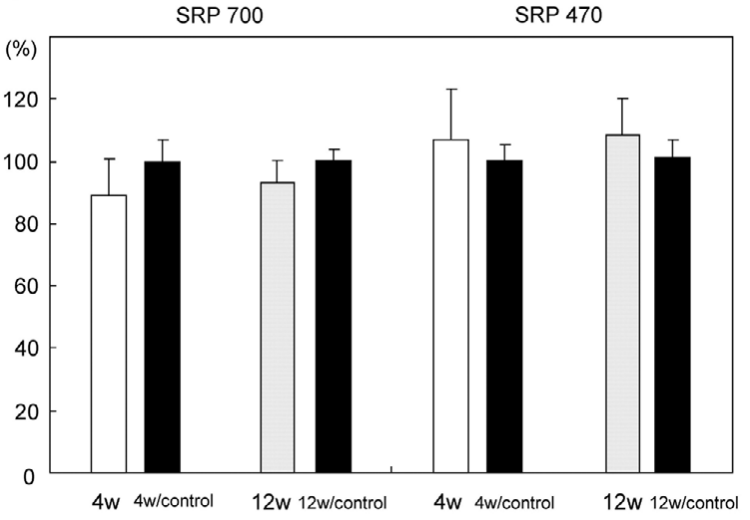
Bar graphs representing the spectral reflectance percentages (SRPs) of the cartilage plugs at 4 and 12 weeks (4 w and 12 w, respectively) after transplantation. The black bar represents the control cartilage for each group. The SRP values are used as a coincidence index of the spectral reflectance distribution of the repaired cartilage with respect to standard intact cartilage. The SRP_700 _values of the cartilage plugs are used as a coincidence index between 400 and 700 nm in wavelength (left four bars), while the SRP_470 _values of the cartilage plugs are used as a coincidence index between 400 and 470 nm in wavelength (right four bars). Error bars represent the standard deviation of each group.

The SRP_700 _values (mean ± standard deviation), as a coincidence index of the spectral reflectance curves, were 89.1 ± 11.6% at 4 weeks and 93.1 ± 7.2% at 12 weeks. There were no significant differences in the SRP_700 _values between the two groups. The SRP_470 _values were 107 ± 16% at 4 weeks and 108.3 ± 11.6% at 12 weeks. There were no significant differences in the SRP_470 _values between the two groups. The SRP_700 _values of the control cartilage were 99.7 ± 7.2% at 4 weeks and 100.3 ± 3.8% at 12 weeks. The SRP_470 _values of the control cartilage were 100.4 ± 5.2% at 4 weeks and 101.5 ± 5.6% at 12 weeks. Compared to the control cartilage, no significant differences were seen in all groups (Figure 2).

### Histological findings

All osteochondral plugs had united in the subchondral area. Eight cartilage plugs (2 at 4 weeks and 6 at 12 weeks) were thicker than the surrounding intact cartilage, while 7 cartilage plugs (4 at 4 weeks and 3 at 12 weeks) were almost the same thickness as the adjacent intact cartilage. The mean histological scores were 2.0 at 4 weeks and 1.4 at 12 weeks. There was no significant difference between the histological scores at 4 and 12 weeks (*P *= 0.51).

**Figure 3:**
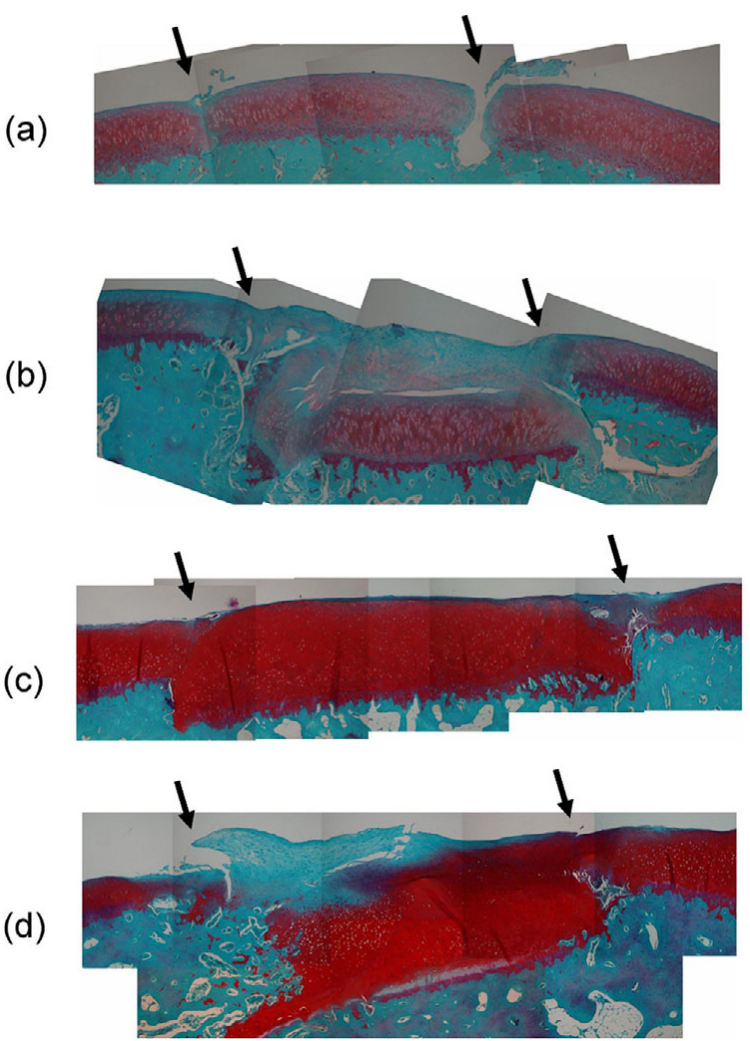
Photomicrographs of cartilage plugs (between the arrows). **(a,b) **At four weeks postoperatively, three plugs were still flush with the surrounding cartilage and retained the hyaline cartilage characteristics (a), while three plugs had sunk or become tilted and their surfaces were covered with newly formed fibrocartilaginous tissue (b). **(c,d) **At 12 weeks postoperatively, 6 plugs retained the hyaline cartilage characteristics (c), while 3 plugs had sunk or become tilted and their surfaces were covered with fibrous tissue (d). Safranin-O fast-green staining; original magnification ×2.5.

From the histological findings, the samples were divided into two groups. Specifically, 9 samples (3 at 4 weeks and 6 at 12 weeks) were classified into group H (Figure 3a,c) and 6 samples (3 at 4 weeks and 3 at 12 weeks) were classified into group F (Figure 3b,d). The histological findings of group H revealed that the cartilage plugs were inserted flush with the surrounding articular surface and maintained the characteristics of hyaline cartilage. The histological findings of group F showed that the cartilage plugs had sunk or become tilted below the level of the surrounding articular surface and their surfaces had become covered with fibrous tissue and/or fibrocartilage. The mean histological scores were 0.6 in group H and 3.3 in group F. There was a significant difference between the histological scores for groups H and F (*P *= 0.001).

### Spectrocolorimetric findings for groups H and F

The histological differences in the L*, a* and b* values of the cartilage plugs and the control cartilage are shown in Table 4. The L* values were significantly lower in group H than in group F (*P *= 0.02), whereas the a* values were significantly higher in group H than in group F (*P *= 0.006). However, the b* values in groups H and F did not differ significantly (*P *= 0.16). Compared to the control cartilage, the cartilage plugs in group H had significantly lower L* and b* values and higher a* values (*P *= 0.001, *P *= 0.003 and *P *= 0.003, respectively) and the cartilage plugs in group F had significantly lower b* values (*P *= 0.003).

**Figure 4:**
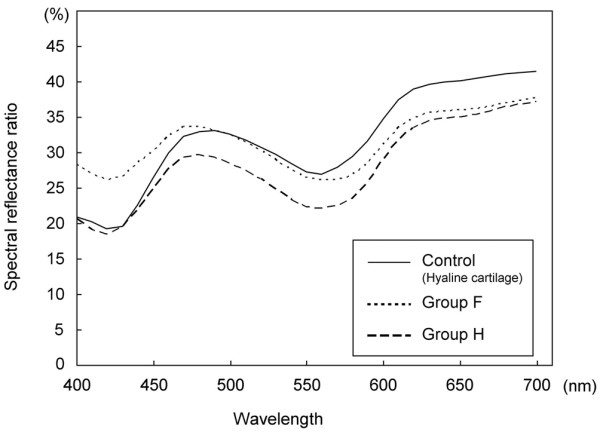
Spectral reflectance curves of groups H and F. Group H consisted of cartilage plugs that retained the features of hyaline cartilage, while group F consisted of cartilage plugs that had become covered with fibrous tissue and/or fibrocartilage. The control samples were intact articular cartilage.

**Figure 5:**
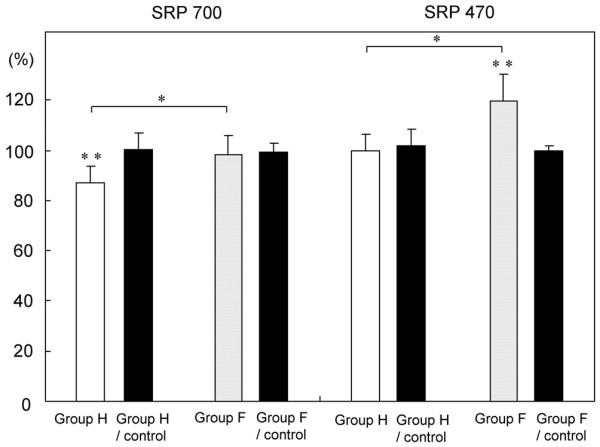
Bar graphs representing the spectral reflectance percentages (SRPs) of groups H and F. The black bar represents the control cartilage for each group. The SRP_700 _values of groups H and F (left four bars) and SRP_470 _values of groups H and F (right four bars) are shown. Error bars represent the standard deviation of each group. *P < 0.05, group H versus group F; **P < 0.05, versus the control; non-parametric Mann-Whitney U-test.

Typical examples of the spectral reflectance curves for control hyaline cartilage, group H and group F are shown in Figure 4. The spectral curves of all the groups showed two dips at 420 and 560 nm and a specific peak around 490 nm. There was a gradual increase in the spectral reflectance ratio from 620 to 700 nm. Across all the measured wavelengths, there was a low reflectance ratio in group H compared with control cartilage. As a characteristic difference, group F had a higher spectral reflectance ratio than control cartilage between 400 to 470 nm. The SRP_700 _values were 86.9 ± 6.7% in group H and 98.4 ± 7.7% in group F, and the difference between these values was significant (*P *= 0.018). The SRP_470 _values were 99.8 ± 6.7% in group H and 119.8 ± 10.6% in group F, and the difference between these values was also significant (*P *= 0.005). The SRP_700 _values of the control cartilage were 100.4 ± 6.3% in group H and 99.5 ± 3.3% in group F. The SRP_470 _values of the control cartilage were 101.9 ± 6.6% in group H and 99.7 ± 2.3% in group F. Compared to the control cartilage, there were significant differences for the SRP_700 _values of group H (*P *= 0.001) and the SRP_470 _values of group F (*P *= 0.004) (Figure 5).

**Table 4:** L* a* b* color change of the plug cartilage in groups H and F

Parameter	Group H	Group F	*P *value^a^
L* value	57.5 ± 2.4^b^	61.2 ± 2.3	*P *= 0.02
L* value/control	62.3 ± 2.0	62.9 ± 2.3	
			
a* value	3.7 ± 0.7^b^	2.4 ± 0.8	*P *= 0.007
a* value/control	2.5 ± 0.8	2.0 ± 1.0	
			
b* value	0.3 ± 1.8^b^	-1.1 ± 1.7^b^	NS
b* value/control	6.2 ± 1.5	6.8 ± 1.4	

Data are mean ± standard deviation. ^a^*P *values based on Mann-Whitney U-test, group H versus F, 12 weeks. ^b^*P *< 0.05 versus control. NS, not significant.

### Relationships between spectrocolorimetric data and the histological score

**Figure 6:**
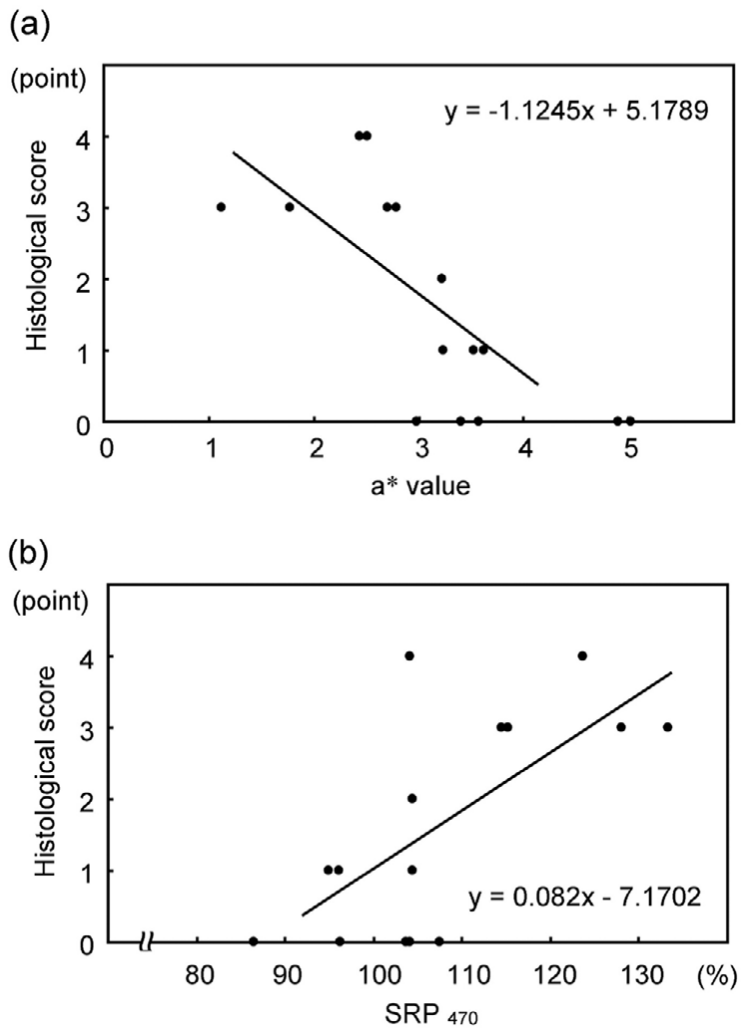
Correlations of the histological scores from microscopic findings and the spectrocolorimetric indices. **(a) **The a* values are significantly correlated with the Mankin histological score (*P *= 0.004, r = -0.76). **(b) **The SRP_470 _values are significantly correlated with the Mankin histological score (*P *= 0.01, r = 0.67). *P *< 0.05 by non-parametric Spearman's rank-order correlation.

The histological score was significantly correlated with the a* values (*P *= 0.004, r = -0.76) and the SRP_470 _values (*P *= 0.01, r = 0.67) (Figure 6), but not correlated with the L*, b* or SRP_700 _values.

## Discussion

In the present study, cartilage plugs after OCG were evaluated quantitatively using the L* a* b* colorimetric system and the spectral reflectance distribution. Our findings demonstrate the ability of spectrocolorimetric measurements to predict the histological findings of cartilage plugs after OCG. In particular, the a* values and SRP_470 _values can be used to judge the surface condition of an osteochondral plug on the basis of objective data.

As new cartilage treatment methods come into use, there will be a requirement for non-invasive evaluation of the regenerated cartilage. A reliable evaluation method, if established, would assist in the solution of the important clinical question regarding the type of cartilage regenerated in lesions, that is, hyaline cartilage or fibrocartilage (fibrous tissue). Magnetic resonance imaging (MRI) may be the most powerful tool for evaluating articular cartilage [14-16]. White and colleagues [16] reported that qualitative and quantitative T2 mapping was useful for differentiating hyaline cartilage from reparative fibrocartilage after cartilage repair using 1.5T MRI. Using optical coherence tomography, Li and colleagues [17] demonstrated real-time imaging of human cartilage in normal and osteoarthritic knee joints. Optical coherence tomography successfully revealed structural changes, including cartilage thinning, fissures and fibrillation, at substantially higher resolutions than those achieved with other currently used clinical imaging technologies. Furthermore, high-frequency ultrasound techniques were recently introduced for detecting the features of regenerated and degenerated cartilage [18-21]. Our previous study revealed that ultrasound analysis can predict the microstructure of regenerated cartilage, and in particular, that this method can differentiate hyaline cartilage from fibrocartilage [22,23]. Laasanen and colleagues [24] further showed that quantitative ultrasound imaging offered diagnostic information regarding the impaired structural integrity of spontaneously repaired cartilage. The present study demonstrates the first spectrocolorimetric assessment of cartilage plugs after OCG.

The application of spectrocolorimetry for medical research is popular in the fields of dermatology and plastic surgery [25-28]. Bohnert and colleagues [25] evaluated subcutaneous bruises after visible contusions in 50 corpses, and found a relationship between the color impression and the localization of the bruise. Li-Tsang and colleagues [26,27] used spectrocolorimetry for scar pigmentation, and found that it showed satisfactory reliability and reproducibility for clinical research. However, there are few published studies of articular cartilage evaluation by spectrocolorimetry. Katayama and colleagues [28] tried to evaluate intact and degenerated meniscuses, while Pilin and colleagues [29] used a digital camera and reported that the color changes of intervertebral discs and rib cartilage were good tools for age estimation.

Based on the findings of the present study, cartilage plugs after OCG can be effectively evaluated by spectrocolorimetry. However, we used the L* a* b* values and SRP values as quantitative indices of the cartilage plugs after OCG, and it is not known what these indices are closely related to. The L* values express the white of the cartilage color, while the a* values express the red of the cartilage color. In the present study, we observed that group H had low L* values and high a* values, whereas group F had high L* values and low a* values. The differences in the L* values imply that the cartilage plug colors differed subtly between the two groups, from translucent (the state of hyaline cartilage) to white (the color of fibrocartilage). Regarding the a* values, several studies on skin color have revealed that the a* values of skin are mainly affected by the degree of blood flow [30,31]. In our cartilage study, the a* values of cartilage should be affected by the blood color of the subchondral bone. Therefore, it is reasonable that the translucent hyaline cartilage had higher a* values than the opaque fibrocartilage.

The spectral reflectance curve represents the most accurate data that can be provided for the characteristics of cartilage color. Therefore, SRP values as a coincidence index for the spectral reflectance distribution were used in the present study. In the dermis, the scattering process of optical radiation due to type I collagen fibers is dominant and skin reflects more light with short wavelengths [25,32]. In the present study, cartilage plugs that maintained a hyaline cartilage surface showed SRP_470 _values of close to 100%, while cartilage plugs that failed to maintain a hyaline cartilage surface and became covered by fibrocartilage and/or fibrous tissue had SRP_470 _values of >100%. Therefore, we suggest that type II collagen is more transparent to light with short wavelengths than type I collagen. Moreover, the SRP_470 _values can provide diagnostically important information about cartilage plugs after OCG. Further detailed research on the assessment of articular cartilage using spectrocolorimetry is now required.

Several limitations of our study should be considered. First, a total cartilage area of 4 mm in diameter could be measured at one time. The measurement area of 4 mm in diameter seems to be too large for the assessment of articular cartilage. Although the proper measurement area should be discussed further, most cartilage defect areas in experimental models are larger than 4 mm in diameter [33-35]. Therefore, spectrocolorimetry is suitable for *in vivo *animal studies. Second, the present machine is too big for use in arthroscopy, and modified machines will be required for clinical assessment of living human cartilage. Finally, the cartilage samples in this study were not human but rabbit cartilage, although spectrocolorimetric assessment of human cartilage is now under investigation.

## Conclusion

The spectrocolorimetric analysis used in the present study was capable of judging the surface condition of an osteochondral plug on the basis of objective data. Therefore, spectrocolorimetry may contribute to orthopedics, rheumatology and related research in arthritis, and arthroscopic use of this method may potentially be preferable for *in vivo *assessment. However, the present machine is too big for use in arthroscopy, and smaller spectrocolorimeters suitable for arthroscopic use are presently being developed.

## Abbreviations

MRI = magnetic resonance imaging; OCG = autologous osteochondral grafting; SRP = spectral reflectance percentage.

## Competing interests

The authors declare that they have no competing interests.

## Authors' contributions

KH conceived the study, participated in its design and performed all the experiments. KU, YT and HY performed the animal study. TH performed the macroscopic and histological assessments. YT performed the histological assessments. YT participated in the design of the animal study.
